# IR beyond the procedure: mastering patient and team management

**DOI:** 10.1186/s42155-025-00592-5

**Published:** 2025-09-29

**Authors:** Sara Lojo-Lendoiro, Jose Andrés Guirola Ortiz, Velio Ascenti, Anna Maria Ierardi

**Affiliations:** 1 Interventional and Vascular Radiology, Hospital Arquitecto Marcide, Ferrol, A Coruña Spain; 2 Interventional and Vascular Radiology, Full Professor Radiology, Hospital Clínico Universitario Lozano Blesa, Zaragoza University, University of Zaragoza, Domingo Miral S/N, 50009 Saragossa, Spain; 3 Diagnostic and Interventional Radiology Department, Fondazione IRCCS Ca`Granda, Ospedale Maggiore Policlinico, Milan, Italia

**Keywords:** Interventional radiology, Multidisciplinary communication, Patient-centered care, Professional role, Health care team, Clinical competence

## Abstract

Interventional Radiology (IR) has evolved far beyond its procedural roots, offering advanced treatments in oncology, vascular disease, and trauma. Its future, however, depends on deeper integration into clinical workflows, patient management, and multidisciplinary collaboration. Barriers such as limited participation in care teams, absence of outpatient consultation structures, and insufficient clinical training hinder IR’s ability to fully own patient care, reducing visibility and delaying referrals. To reframe IR as a true clinical specialty, several actions are needed: expand integration through tumor boards, ward rounds, and follow-up clinics; strengthen patient-centered communication to support trust and informed consent; assume leadership roles to improve workflows, staffing, and institutional decision-making; reform training with broader clinical exposure, simulation, and standardized certification (e.g., EBIR); and establish structural consistency across departments to unify identity and foster collaboration. The future of IR lies in combining technical expertise with patient engagement, interdisciplinary cooperation, and clinical leadership—ensuring it becomes a central, not peripheral, force in modern healthcare. This review will explore the evolving role of interventional radiologists in patient management, with particular emphasis on their integration into hospital-based multidisciplinary teams. It will examine their participation in clinical decision-making, interprofessional communication, and strategies to enhance the visibility and recognition of IR among both patients and healthcare providers.

## Introduction

Interventional Radiology (IR) has experienced a profound transformation in recent years, evolving well beyond its traditional role in image-guided procedures (1). Previously regarded primarily as a technical discipline focused on performing minimally invasive interventions, IR now occupies a central position in the delivery of innovative therapeutic solutions—many of which were once the exclusive domain of surgeons and other procedural specialists.

Advancements in device technology and imaging guidance have empowered interventional radiologists (IRs) to manage increasingly complex clinical scenarios. These range from oncological therapies and vascular interventions to musculoskeletal, hepatobiliary, and emergent trauma care, thereby broadening the spectrum of conditions amenable to minimally invasive treatment. However, this evolution transcends technical capabilities; it is fundamentally reshaping the role of IRs within patient care pathways and healthcare systems at large.

The traditional conceptualization of the IR as a procedural technician fails to reflect the broader scope and responsibility now inherent to the specialty. A paradigm shift toward more patient-centered care demands early involvement of IRs in the clinical process—including direct patient consultations, shared decision-making, and longitudinal follow-up (2). Structured integration into multidisciplinary teams (3), as well as the development of outpatient consultation models and direct referral mechanisms, are essential to fully realize the clinical potential of IR and to optimize patient outcomes.

As the specialty continues to expand, a critical challenge lies in redefining its perception within the medical community and ensuring that healthcare institutions support and institutionalize this broadened role. Embracing this evolution will enable IRs to contribute more meaningfully to comprehensive care delivery and to offer innovative, patient-centered solutions grounded in minimally invasive therapy.

Despite significant progress, many patients remain unaware that interventional radiologists can be consulted directly to explore therapeutic alternatives. Efforts to increase awareness—through public education campaigns, ongoing professional development initiatives, and a stronger presence in outpatient care settings—are imperative to ensure timely access to IR services and to minimize unnecessary referrals or delays.

This review will explore the evolving role of interventional radiologists in patient management, with particular emphasis on their integration into hospital-based multidisciplinary teams. It will examine their participation in clinical decision-making, interprofessional communication, and strategies to enhance the visibility and recognition of IR among both patients and healthcare providers.

## The current role of IRs

### Lack of clinical training and participation in work teams

Despite the increasing therapeutic potential of interventional radiology, in most healthcare systems around the world, the role of the IRs is still largely confined to procedural work. In fact, although in many institutions interventional radiologists actively participate in multidisciplinary team (MDT) meetings where treatment strategies are defined collaboratively, once a consensus is reached and the procedure is performed, IRs are often excluded from subsequent phases of care. As a result, patients might encounter their interventional radiologist for the first—and sometimes only—time on the day of the procedure [4]. This confirms the idea that in everyday clinical practice, until IRs are integrated into the full cycle of patient care, from initial evaluation to long-term follow-up, the specialty will remain at the margins of clinical medicine and IR professionals will keep being perceived as “proceduralists” rather than full “practitioners”[5].

This fragmentation contributes significantly to the specialty’s limited recognition among patients and also undermines the specialty’s authority within the healthcare system. One of the key factors in this limited integration is IR’s historical alignment with diagnostic radiology, a field with minimal clinical exposure. In addition, traditional IR pathways prioritize technical skills while neglecting clinical decision-making, longitudinal patient care and lack rotations in core medical and surgical specialties, leaving trainees less equipped to function as independent clinicians [6].

Another limit in the development of IR as a clinical specialty is the lack of outpatient access and formal consultation channels. In fact, in many institutions, IRs don’t have an ambulatory patient list, making it difficult for patients or referring physicians to book consultations directly [7].

To overcome these problems, a few propositions have been made. For example, several countries like the United States, Canada, UK, and Japan have revised residency programs to incorporate rotations in internal medicine, surgery, and critical care in order to strengthen the clinical foundation of IR training, and to achieve that future specialists can assume responsibility for peri-procedural and long-term patient management [8, 9].

In high-functioning IR departments, however, this model is already being actively pursued. A mature interventional radiology unit should operate with distributed roles: while some team members focus on procedural work, others should conduct ward consultations, manage outpatient clinics or perform follow-up on patients previously treated [10]. Such organizational structures not only enhance workflow efficiency but also reinforce the clinical presence of IRs within the hospital ecosystem. Effective teamwork should not be limited to physician collaboration but also by interprofessional (IP) dynamics, involving radiographers, nurses, and other healthcare professionals: communication quality, hierarchical flexibility and shared situational awareness are all crucial to ensuring safe and efficient patient care [11].

### Competition with other specialties for procedures (and its influence on relationships between the different teams)

An IR is, at their core, a medical specialist who uses imaging guidance to perform minimally invasive procedures. Over the last decades, IRs have been pioneers of therapeutic innovation, developing procedures that are now considered standard of care in the treatment of numerous pathologies like peripheral arterial disease, hepatocellular carcinoma’s, renal and biliary obstruction, and many others. [12]

However, the widespread success of these techniques has also made them attractive to other clinical specialties, like, among others, vascular surgeons, cardiologists and urologists, who are now performing procedures that once fell almost exclusively under the domain of IR. One of the oldest ‘turf wars’ between various medical specialists seems in fact to be that between interventional radiologists and vascular surgeons. Keller et al., attempted to analyse this competition scientifically by interviewing several radiologists and surgeons and analysing their responses; they found out that despite the common notion that money and ego can certainly exacerbate interdisciplinary tension, cultural differences and different mindsets between specialties played the most important role in both competition and collaboration. In fact, it seems that interventional radiologists tend to view themselves as procedure experts, while vascular surgeons see themselves as disease experts. [13] Levin et al. in 2005 argued that radiologists were the most experienced and best-trained professionals for peripheral non-cardiac vascular interventions, suggesting that procedural ownership should remain within IR to maintain care quality. [14] However, over the past two decades, that argument has aged poorly since today technical skills are reproducible through robust training. What truly determines procedural access is clinical integration and patient referral pathways. In addition, since many of these patients are managed primarily by other clinicians, IRs often intervene only when patients are referred explicitly or when other options are exhausted, because other specialists have deemed them inoperable, too high-risk for surgery or out of options. [15] This late-stage referral pattern diminishes IR’s impact and reinforces a perception of the specialty as a parachute solution rather than a primary treatment provider. Technical proficiency is no longer sufficient; it is clinical integration and ownership of the therapeutic strategy that determines influence. [10] Nonetheless, the solution is not to monopolize procedures.

These confrontations stem from territorial disputes, credentialing conflicts, differing procedural approaches, and economic pressures [16].

Beyond procedural innovation, one of IR’s most distinctive contributions to modern healthcare lies in its ability to deliver effective care in outpatient settings. This ability to provide outpatient procedures and dedicated ambulatory care services is one of interventional radiology’s most distinctive advantages. Many image-guided interventions can be performed safely without hospital admission, using minimally invasive techniques that minimise anaesthetic risk, reduce procedural morbidity, and allow same-day discharge. This approach shortens recovery times, lowers healthcare costs, and improves patient convenience, while maintaining high standards of safety and efficacy. Outpatient IR services also facilitate rapid access to care, streamline follow-up, and strengthen the specialty’s role as a primary therapeutic provider rather than a secondary referral option.

To resolve tensions, institutions should promote multidisciplinary collaboration, establish clear credentialing standards, and enhance IR visibility through education and outreach. Focusing on patient-centered care rather than turf battles can transform competition into cooperation, ultimately leading to better outcomes and a more integrated healthcare approach. Interdisciplinary collaboration must remain central, and IR should aim to contribute its strengths within integrated care models. A patient-first approach—where specialties cooperate rather than compete—is the most effective way to ensure high-standard quality of care.

### SWOT analysis

IR stands out as a recognized clinical specialty with a solid foundation in oncology, vascular, and trauma care, backed by active professional societies like CIRSE and SIR. Its strengths include high patient demand for minimally invasive procedures, proven value within multidisciplinary teams, and technological advances enhancing precision and safety. However, IR faces internal challenges such as fragmented training, variable clinical involvement, limited awareness of its full scope, and inconsistencies in credentialing. Opportunities lie in developing unified training pathways, competency-based certification, expanding into novel therapeutic areas, and adopting digital health tools. Yet, threats such as turf encroachment from other specialties, financial and regulatory hurdles, technological disruption, and workforce strain pose significant risks to sustained growth and recognition.

You can see the SWOT analysis on Table 1.

**Table 1:** SWOT analysis

Strengths	Weaknesses	Opportunities	Threats
Recognition as a clinical specialty	Fragmented training and non-standardized residency	Integrated IR-DR residency tracks	Encroachment by other specialties
High demand for minimally invasive procedures	Limited longitudinal patient management	Standardized competency-based certification	Reimbursement and regulatory issues
Efficacy in multidisciplinary care	Low public and intra-hospital awareness	Expansion into emerging therapeutic areas	Technological disruptions
Advancements in imaging technologies	Variable credentialing across regions	Use of digital/telemedicine platforms	Workforce shortages and burnout

### What needs to change:

Changes are needed to maximize the impact of the profession and enhance patient care. You can visualize the integration of the four axes of change in Table 2.

**Table 2:** Key areas for strengthening the clinical role of the IR

Area	Integration	Communication	Leadership	Training
Definition	Embed the IR into diagnosis, treatment and follow up processes	Promote patient-centered care and effective interprofessional relationships	Position the IR as a key actor in clinical, organizational, and educational decisions	Ensure comprehensive clinical and technical training based on competencies
Current weakness	• “Proceduralist” perception of the IR • Lack of outpatient clinics	• Limited training in communication skills • Lack of awareness of the IR role	• Little participation in hospital committees • Limited staffing • Professional burnout	• Traditional training focus on technique • Lack of clinical rotations • Lack of standards
Proposal for improvement	• Active participation in multidisciplinary boards (e.g. tumor boards, PERT) • Dedicated IR clinics	• Patient education • Improved informed consent • Interdisciplinary and empathic communication	• Access to management positions • Advocacy for adequate staffing	• Integrated clinical rotations (medicine, ICU, surgery) • Simulation • European standard EBIR

### Greater participation in multidisciplinary teams or tumor boards:

In modern healthcare, IR cannot exist in isolation. The complexity of today’s patient care demands that IRs are fully integrated members of multidisciplinary teams, not peripheral consultants [17]. Active collaboration is no longer optional — it is essential to delivering the best outcomes.

Pulmonary Embolism Response Teams (PERTs) exemplify how IR’s involvement at the core of multidisciplinary decision-making transforms patient care. In the management of high-risk pulmonary embolism, timely intervention often makes the difference between life and death. When IRs work alongside cardiologists, pulmonologists, intensivists, and hematologists, patients benefit from earlier diagnosis, expanded treatment options such as catheter-directed therapies, and faster access to life-saving interventions [18, 19]. Without full integration into the team, IR’s capabilities risk being underutilized, and patient care suffers.

Similarly, the presence of interventional radiologists in oncologic tumor boards is critical to ensuring that patients are considered for minimally invasive treatments that can offer curative or palliative benefits. It is undeniable that the future of IR lies in areas such as embolisation, palliative care and oncology patient management. Studies demonstrate that when IR is consistently present during tumor board discussions, there is better alignment of imaging, diagnosis, and therapeutic options, leading to more personalized and often less invasive care pathways. In this setting, interventional radiologists are not just technicians — they are clinical decision-makers whose expertise can change the course of treatment (17,20).

Being a true team member also fosters earlier referrals, greater trust among specialties, and a deeper understanding of how IR can contribute throughout the continuum of care — from diagnosis to treatment to follow-up [21–23]. Importantly, it elevates the visibility and value of the specialty within the hospital ecosystem.

Today, being part of a multidisciplinary team is not simply beneficial for interventional radiology — it is indispensable. Only by embedding ourselves fully into these collaborative structures can we ensure that our patients receive the highest standard of care.

### Promoting patient-centered care and communication

As IR evolves into a more patient-facing specialty, integrating patient-centered care and communication skills has become essential. IRs must embrace their role not only as procedural experts but also as communicators, educators, and advocates for their patients (24).

A key aspect of promoting patient-centered care is proactive patient education. IRs should take the time to clearly explain procedures, potential risks, expected benefits, and alternative options. However, patient education should go beyond procedural details: it must also include setting realistic expectations for recovery, pain management, and possible long-term outcomes. Studies have shown that patients who are well-informed before procedures experience lower levels of anxiety, demonstrate better adherence to follow-up recommendations, and report higher satisfaction with their care [25].

Moreover, effective communication in IR requires emotional intelligence — the ability to perceive, understand, and respond to patients’ emotions and concerns. Before, during, and after procedures, patients may experience fear, uncertainty, or confusion. Demonstrating empathy, active listening, and clear, jargon-free explanations helps build trust and can significantly impact a patient’s overall experience [26]. These skills are particularly important during informed consent discussions, where patients may be overwhelmed by complex information and need emotional support as much as technical clarity. Recent literature also highlights that a patient-centered approach improves not only patient satisfaction but also procedural outcomes. When patients feel heard and involved in their care plans, they are more likely to engage in shared decision-making and to adhere to postoperative instructions, which can reduce complications and readmissions [27, 28].

Beyond technique, longitudinal contact with patients allows IRs to practice more humane medicine, based on trust, empathy and continuity of care. This bond, which is built from the initial consultation to the post-procedure follow-up, improves the patient’s experience, reinforces shared decision-making and gives the IR a more visible and valued role in the therapeutic journey. The humanization of care is not an add-on, but a powerful clinical tool to improve outcomes.

Incorporating patient-centered communication into daily IR practice requires formal training, deliberate practice, and a cultural shift within departments that recognizes communication as a core clinical skill. By prioritizing patient education, emotional intelligence, and compassionate communication, IRs can enhance the quality of care and solidify their role as integral, trusted members of the healthcare team.

### Expanding leadership roles

There is an urgent need to expand the leadership roles of IRs within healthcare institutions: traditionally viewed as procedural specialists, IR professionals are uniquely positioned to contribute far beyond the angiography suite. By increasing their visibility and influence in hospital decision-making processes, interventional radiologists can help shape more efficient workflows, optimize resource allocation, and drive improvements in patient care practices across multiple departments.

Admission and billing rights are fundamental to interventional radiology’s evolution into a true clinical specialty. Without the ability to admit patients directly, IRs remain dependent on other services for bed allocation and inpatient management, which limits timely intervention and continuity of care. Independent billing rights ensure recognition of the full clinical episode—from pre-procedure evaluation to post-procedure follow-up—rather than just the technical component. Securing these rights strengthens IR’s role in the therapeutic pathway, enables direct patient relationships, and aligns incentives toward comprehensive, patient-centered care.

A key strategy is to actively seek leadership positions within hospital committees, multidisciplinary teams, and administrative boards. By participating in these structures, IRs can advocate for evidence-based, minimally invasive solutions that benefit patients and reduce overall healthcare costs. Furthermore, being at the table when decisions are made allows IRs to address logistical bottlenecks, advocate for necessary technological investments, and streamline patient referral pathways, ultimately improving both departmental performance and patient outcomes (29).

Expanding leadership roles also means taking a broader view of patient care. Resident doctors must be more directly involved in the physician's clinical management, follow-up and patient education. Taking responsibility for the entire patient journey strengthens relationships with both patients and referring physicians, reinforcing the essential role of residents in modern healthcare.

In addition, leadership in education and research should not be overlooked. It is essential to be present in science with studies that support our activity, encouraging high evidence clinical studies that demonstrate not only that we work hard, but also that we do it with all the quality standards.

It's important to note that increasing demand for IR procedures highlights the urgent need to expand and reinforce IR teams. Performing complex, high-risk procedures alone is not only unsafe but also unsustainable. Adequate staffing is essential to ensure patient safety, maintain procedural quality, and protect the physical and mental health of IR physicians. Working alone or being heavily outnumbered by procedure requests leads to fatigue, rushed interventions, higher complication rates, and burnout. To meet growing clinical needs, IR departments must advocate for more trained personnel — including IR physicians, nurses, and technologists — ensuring that no interventionalist faces critical procedures without proper team support.

Prioritizing team growth is not a luxury; it is a fundamental requirement for delivering safe, effective, and resilient interventional radiology services.

Ultimately, expanding leadership roles will require proactive communication, interdisciplinary collaboration, and a willingness to step outside traditional boundaries. IRs must position themselves not only as technical experts but as strategic partners in the broader mission of delivering high-quality, patient-centered healthcare, but we must not forget that avoiding burnout and maintaining mental and occupational health is necessary.

### From Babel to blueprint: the urgent need for unity in interventional radiology

IR urgently needs greater homogeneity—both in the standardization of procedures and techniques, and in the structural organization of its services across hospitals. Despite being a recognized specialty, IR still functions like a fragmented ecosystem, with wide variability in how care is delivered, what roles interventional radiologists assume, and how departments are structured within institutions. This inconsistency creates confusion not only among referring physicians and hospital administrators but also within the specialty itself, weakening its collective identity and impact. The current state of IR can be likened to the Tower of Babel: a group with a shared purpose but speaking different “languages”—each unit or hospital developing independently, leading to miscommunication, inefficiencies, and missed opportunities for unified growth. To avoid this architectural collapse, IR must strive toward a shared framework that aligns training, procedures, clinical involvement, and departmental models. Only through coherence can the specialty reach its full potential and speak with one powerful, coordinated voice.

### Improved training

To fully realize the potential of interventional radiology as a clinical specialty, training programs must prepare new generation of IRs not only with technical skills but also as clinicians capable of managing patients before and after procedures. In a recent publication, Morgan et al. defined the clinical scope of the IRs, highlighting that IRs should be responsible for patient consultation, procedure planning, execution, and follow-up, similarly to surgical and medical specialties.

The paper also underlines that IRs must have dedicated training pathways, independent certification processes and recognition by health authorities as a standalone specialty. [9]

A paradigm shift has already begun to revolutionize the traditional training pathway in the United States, where a previously comprised program of a 1-year vascular and IR fellowship after diagnostic radiology residency, has been modified with the introduction of integrated and independent IR residencies, which integrate diagnostic radiology with IR and makes mandatory clinical rotations such as in surgery, internal medicine, intensive care unit. Alongside the United States, many countries like the United Kingdom, Canada, Japan, Australia and New Zealand are pursuing significant reforms in interventional radiology training programs with emphasis on direct patient contact [4, 9, 30, 31].

For what concerns technical skills, simulation training has also been identified as a valuable tool to increase confidence and improve skills among trainees and inexperienced operators. Kreiser et al. shared their experience with an angiography simulator integrated within the angiography suite. According to the authors, such a system can enhance the performance of less experienced operators and students, making it a very interesting tool for both education and research. [32]

In parallel, the digital transformation of medicine offers a key opportunity to integrate IRs more effectively into the clinical lifecycle. Interoperability between systems such as HIS, RIS and PACS allows IRs to access comprehensive patient information, document consultations, generate longitudinal reports and actively participate in clinical follow-up. Good digital integration facilitates the implementation of IR outpatient consultations, improves communication with other specialists and positions the IR as a visible and accessible physician.

### Advancing interventional radiology through CIRSE initiatives

The European Society of Cardiovascular and Interventional Radiology (CIRSE) carries out interesting initiatives, such as the standardisation of training programmes in interventional radiology and, consequently, the level of clinical competence, through the use of an internationally organised examination, the European Board of Interventional Radiology (EBIR), as well as the EBIR-ES and EBIR-IO certifications (certifications in endovascular subspecialisation and interventional oncology, respectively) [33–35]. These certifications not only validate procedural expertise but also reinforce the broader clinical competencies essential to comprehensive patient care. Beyond education, CIRSE has created multiple working groups and teams that address critical aspects of the specialty, such as patient safety, radiation protection, sustainability in IR practice, and the integration of outpatient services.

Also noteworthy are the society's committees and subcommittees, which focus on identifying systemic challenges, fostering multidisciplinary collaboration, and promoting the inclusion of IR procedures in clinical guidelines. Through position statements, consensus documents, and quality improvement initiatives, these groups actively shape standards of care and advocate for the visibility of IR within the broader healthcare system.

All of these initiatives are available and can be explored on the CIRSE website (36).

## Conclusion

The future of interventional radiology depends on more than technical skill. To deliver high-quality, safe care, IRs must be fully engaged in patient management, multidisciplinary collaboration, and scientific validation of their work. Strengthening clinical training, increasing visibility within care teams, and reinforcing IR staffing are essential steps to avoid procedural isolation and ensure sustainable growth.

To solidify its clinical role, IR must also define and track meaningful impact indicators—such as reduced hospital stay, complication rates, and patient satisfaction—to demonstrate measurable value. Finally, by embracing leadership in clinical care, research, and team coordination, IR can continue to expand its role and improve outcomes across the healthcare system.

## Data Availability

Not applicable.

## Abbreviations

IR: Interventional Radiology
IRs: Interventional Radiologists
MDT: Multidisciplinary Team
PERT: Pulmonary Embolism Response Team
ICU: Intensive Care Unit
HIS: Hospital Information System
RIS: Radiology Information System
PACS: Picture Archiving and Communication System
EBIR: European Board of Interventional Radiology
CIRSE: Cardiovascular and Interventional Radiological Society of Europe
SIR: Society of Interventional Radiology
IP: Interprofessional
SWOT: Strengths, Weaknesses, Opportunities, Threats
